# School-level electronic cigarette use prevalence and student-level tobacco use intention and behaviours

**DOI:** 10.1038/s41598-018-38266-z

**Published:** 2019-02-08

**Authors:** Jianjiu Chen, Sai Yin Ho, Lok Tung Leung, Man Ping Wang, Tai Hing Lam

**Affiliations:** 1 School of Public Health, University of Hong Kong, Hong Kong, People’s Republic of China; 2 School of Nursing, University of Hong Kong, Hong Kong, People’s Republic of China

## Abstract

Prevalent electronic cigarette (e-cigarette) use in schools may undermine tobacco denormalisation, and thus increase tobacco use in students. We investigated the associations of school-level e-cigarette use prevalence with student-level intention and behaviours related to e-cigarettes, cigarettes, and other tobacco products. In a 2014-15 school-based cross-sectional survey of 41035 secondary school students (grade 7–12; age 11–18 years) in Hong Kong, information was collected on the use of e-cigarettes, cigarettes, and non-cigarette tobacco products (NCTPs), susceptibility to e-cigarette and cigarette use, intention to quit cigarette smoking, and sociodemographic characteristics. The adjusted odds ratio (AOR) of e-cigarette use susceptibility in relation to high (vs low) school-level e-cigarette use prevalence was 1.40 (95% CI 1.05–1.87) in never e-cigarette users. The AORs of cigarette smoking susceptibility in relation to medium and high (vs low) school-level e-cigarette use prevalence were 1.24 (1.01–1.52) and 1.34 (1.02–1.75), respectively, in never cigarette smokers. School-level e-cigarette use prevalence was associated with ever and past 30-day cigarette smoking, but not with intention to quit (in past 30-day cigarette smokers) or past 30-day NCTP use. The findings highlight the importance of strictly banning e-cigarettes in schools, and add to the evidence that prevalent e-cigarette use in adolescents may increase cigarette smoking prevalence.

## Introduction

Electronic cigarettes (e-cigarettes) have been embraced by some as a harm reduction product that can reduce cigarette consumption and increase cessation^1,2^. Others, however, argue that e-cigarettes impede smoking cessation^3^. In addition, although e-cigarettes are considered less harmful than cigarettes, research suggests that e-cigarettes’ harm may still be substantial, including cancers and cardiovascular and respiratory diseases^3^.

In many countries, e-cigarettes have gained popularity in adolescents. Past 30-day e-cigarette use prevalence increased from 1.5% in 2011 to 20.8% in 2018 in United States (US) high school students^4^; and from 5.5% in 2011 to 29.9% in 2014 in Poland high school students^5^. Past 30-day e-cigarette use was also prevalent in Canadian (9.0% in 2014/15) and Korean (4.7% in 2011) adolescents^6,7^. In Hong Kong, the 2014/15 School-based Survey on Smoking (SSS; on which the present study was based) found a past 30-day e-cigarette use prevalence of 4.0% in secondary school students (grade 7–12).

As adolescents spend a large amount of time in schools, a school environment with prevalent e-cigarette use may encourage e-cigarette uptake in never e-cigarette users. This was explored in only one cross-sectional study in the US^8^. The study found that high school-level prevalence of e-cigarette use was associated with intention to use e-cigarettes in never e-cigarette users. However, whether the finding can be replicated in vastly different settings is unclear.

More importantly, it is possible that e-cigarettes’ popularity in schools can undermine the denormalisation of tobacco in general and even increase the use of conventional tobacco products, including cigarettes. Indeed, an e-cigarette company advertising officer has spoken explicitly about their expectation for such “renormalisation”^9^. It is therefore also necessary to investigate the association of school-level e-cigarette use prevalence with student-level intention and behaviours related to cigarettes and other tobacco products. But we found no such study. A closely related issue is the gateway theory in which individual-level e-cigarette use leads to cigarette smoking as supported by a recent meta-analysis of cohort studies^10,11^. E-cigarettes’ reversal of tobacco denormalisation is believed to have a role in the gateway effect, along with other mechanisms^10^.

Decades of efforts in tobacco control have reduced Hong Kong’s adult daily cigarette smoking prevalence to 10.0% in 2017^12^, which was among the lowest in the world. In Hong Kong secondary school students, the past-30 day cigarette smoking prevalence was 4.8% in the SSS 2014/15. Any renormalisation of tobacco through new products such as e-cigarettes would threaten to halt or reverse the progress made. Therefore, investigation of school-level and student-level e-cigarette use’s effect on conventional tobacco product use will both have important implications for tobacco control.

We used cross-sectional data of Hong Kong secondary school students from the SSS 2014/15 to investigate the associations of school-level e-cigarette use prevalence (referred to as “school-level e-cigarette use” below) with student-level (a) e-cigarette use susceptibility; (b) cigarette use susceptibility and behaviours and intention to quit cigarette smoking; and (c) non-cigarette tobacco product (NCTP) use.

## Methods

### Data source

The SSS 2014–15 was a territory-wide cross-sectional survey in Hong Kong secondary school students (grade 7–12). A probability sample of schools stratified by districts was used. All students in the recruited schools were invited. Anonymous paper-and-pencil questionnaires were administered to students in classrooms by teachers who followed standard procedures on an instruction sheet. Trained student research assistants were also sent to schools, with 1 assistant per grade, to help answer students’ queries. To limit the length of our questionnaires, three versions were used, each comprising core questions (common for all versions) and version-specific questions. Each recruited school was randomly assigned one version of questionnaire. The questions covered sociodemographic characteristics; tobacco-related knowledge, attitude, intention, and behaviours; and other health-related behaviours. Each questionnaire took 20 to 30 minutes to complete.

Before the survey, invitation letters were sent to parents via students. Parents who did not want their children to participate were to ask their children to return a blank questionnaire after the survey. Student participation was voluntary even with parental permission. Written informed consent was not used. Ethics approval of this survey was granted by the Institutional Review Board (IRB) of the University of Hong Kong/Hospital Authority Hong Kong West Cluster; all survey procedures were conducted in accordance with those approved by the IRB.

Completed questionnaires were collected from 41035 students in 92 schools, with student- and school-level response rates of 95% and 36%, respectively. The questionnaires with missing data for age, sex, or over 50% of items (n = 468) and those with multiple internal logical inconsistencies (n = 365) were excluded, leaving 40202 for analysis. The analyses on NCTPs used a subsample of 10923 students from 30 schools who provided such data.

The age of Hong Kong secondary school students generally ranged from 11 to 18 years. In 2013, 65.7% of adult daily cigarette smokers in Hong Kong reported that they became weekly smokers by the age of 19^13^. Secondary school students are, therefore, of critical importance in smoking prevention, and were selected for the present investigation.

### Measurements

Susceptibility to e-cigarette use and cigarette smoking were both assessed by two questions: “Do you think you will use e-cigarettes (or smoke cigarettes) in the next 12 months?” and “If one of your good friends offers you an e-cigarette (or a cigarette), will you use (or smoke) it?” These questions had 4 options: “Definitely not”, “probably not”, “probably yes”, and “definitely yes”. For a given product (e-cigarettes/cigarettes), the students who chose “definitely not” for both questions were deemed not susceptible to use that product; otherwise they were deemed susceptible to use that product. The susceptibility measures of e-cigarette and cigarette use are both strong predictors of future use of the respective product^14,15^.

Ever e-cigarette use and cigarette smoking (both described as even a puff) were reported (yes/no). Past 30-day e-cigarette use and cigarette smoking were also reported, with the responses recoded into “yes” (1–2 days/3–5/6–9/10–19/20–29/30) and “no” (0 day). Current intention to quit cigarette smoking was reported (yes/no). Past 30-day use of NCTPs, including waterpipe, chewing tobacco, cigar, snus, smoking pipe, and other tobacco products, were also reported (yes/no).

Alcohol drinking was assessed by one question: “How often do you drink alcohol or alcoholic beverages?” with options of (a) I don’t drink; (b) less than 1 day per month; (c) 1–3 days per month; (d) 1–3 days per week; (e) 4–6 days per week; and (f) daily. Monthly drinking was defined as choosing c-f (vs a-b); weekly drinking, d-f (vs a-c); daily drinking, f (vs a-e). Perceived family affluence was assessed by one question: “You consider your family’s economic status:” with options of “relatively poor”, “poor to average”, “average”, “average to rich”, and “relatively rich”. Sex and age (in years) were also reported.

### Analysis

Descriptive analyses were weighted by age, sex, and grade based on the target population’s characteristics provided by the Education Bureau of the Hong Kong Government. Correlations of school-level past 30-day e-cigarette use with school-level prevalence of other intention and behaviour indicators related to tobacco use were estimated using Pearson’s correlation coefficients (rs).

Two-level logistic regression models with a random school-level intercept were used to investigate associations. The study factor, in main analysis, was school-level past 30-day e-cigarette use, analysed as both a 3-level and a continuous variable. Schools were evenly divided into these 3 levels that, respectively, had low (reference), medium, and high prevalence of past 30-day e-cigarette use. The associations with susceptibility to e-cigarette use and cigarette smoking (outcomes) were investigated in never e-cigarette users and never cigarette smokers, respectively. The associations with ever and past 30-day cigarette smoking (outcomes) were investigated in all; the association with intention to quit (outcome) was investigated in past 30-day cigarette smokers. In the subsample of 10923 students from 30 schools, the associations with past 30-day NCTP use (outcomes) were investigated in all. In sensitivity analysis, all main regression analyses were repeated with the study factor replaced by school-level ever e-cigarette use.

Each association of interest was investigated in a crude and an adjusted model. All adjusted models included age, sex, and perceived family affluence as covariates. Additional covariates included cigarette smoking for e-cigarette use susceptibility (outcome), and e-cigarette use for all outcomes related to cigarettes. For NCTP use (outcomes), both e-cigarette use and cigarette smoking were adjusted for. For all outcomes except ever and past 30-day cigarette smoking, school-level past 30-day cigarette smoking prevalence (continuous) was adjusted for.

Negative control analysis, formalised by Lipsitch *et al*., was used to explore confounding bias^16^. The associations of school-level past 30-day e-cigarette use (study factor) with monthly, weekly, and daily alcohol drinking (negative controls) were investigated. The analyses were adjusted for age, sex, perceived family affluence, e-cigarette use, cigarette smoking, and school-level past 30-day cigarette smoking prevalence (continuous). Drinking generally met the two selection criteria for negative controls. First, drinking (negative control) was not plausibly affected by the study factor (school-level past 30-day e-cigarette use). Second, if unmeasured confounders induced spurious associations between the study factor and the outcomes of interest, the same confounders should likewise have affected the association between the study factor and drinking. This was plausible because of the significant overlap in the upstream determinants of drinking, cigarette smoking, and other tobacco use^17^. Therefore, if the study factor was associated with both the outcomes of interest and drinking, this would suggest confounding effect on all observed associations; if associations were observed only for the outcomes of interest, this would add credence to causal interpretations. All analyses used Stata version 13.1.

## Results

Table 1 shows that 9.3% of students in the whole sample (n = 40202) were ever e-cigarette users; 4.0% were past 30-day users. Ever and past 30-day cigarette smoking prevalence were 13.2% and 4.8%, respectively. In never e-cigarette users, 10.3% were susceptible to e-cigarette use; in never cigarette smokers, 10.1% were susceptible to cigarette smoking. In past 30-day cigarette smokers, 28.9% had an intention to quit. In the subsample (n = 10923), the prevalence of past 30-day use of NCTPs were generally low (Table 2): Waterpipe (1.3%), chewing tobacco (0.3%), cigar (0.6%), snus (0.4%), smoking pipe (0.3%), and other tobacco products (0.4%). In the whole sample (92 schools), school-level past 30-day e-cigarette use ranged from 0% to 18.3%; school-level ever e-cigarette use, 0% to 50.0%. In the subsample (30 schools), the corresponding figures were 0% to 12.6% and 0% to 21.4%.

**Table 1:** Sample characteristics (n = 40202).

	N^a,b^	%^b^
**Sex**		
Girls	19489	48.5
Boys	20713	51.5
**Mean age in years (standard deviation)**	14.9 (1.8)	
**Perceived family affluence**		
Relatively poor	2459	6.1
Poor to average	9324	23.3
Average	22049	55.0
Average to rich	5294	13.2
Relatively rich	959	2.4
**E-cigarette use**		
Never	36334	90.7
Ever, not past 30-day	2121	5.3
Past 30-day	1615	4.0
**Cigarette smoking**		
Never	34808	86.8
Ever, not past 30-day	3395	8.5
Past 30-day	1908	4.8
**E-cigarette use susceptibility (in never e-cigarette users)**		
No	32528	89.7
Yes	3739	10.3
**Cigarette smoking susceptibility (in never cigarette smokers)**		
No	31157	89.9
Yes	3493	10.1
**Intention to quit (in past 30-day cigarette smokers)**		
No	1358	71.2
Yes	551	28.9

^a^Numbers unless otherwise stated.

^b^Numbers and proportions were weighted by age, sex, and grade based on the target population’s characteristics provided by the Education Bureau of the Hong Kong Government.

**Table 2:** NCTP use prevalence^a^.

	N^b^	%^b^
**NCTPs**		
Waterpipe	145	1.3
Chewing tobacco	32	0.3
Cigar	70	0.6
Snus	42	0.4
Smoking pipe	33	0.3
Other tobacco products	44	0.4

^a^NCTP = Non-cigarette tobacco product. The prevalence was estimated in a subsample (10923 students; 30 schools) of the whole sample (40202 students; 92 schools). Only the subsample had data on NCTP use.

^b^Numbers and proportions were weighted by age, sex, and grade based on the target population’s characteristics provided by the Education Bureau of the Hong Kong Government.

Supplementary Fig. 1 shows that the Pearson’s rs of school-level past 30-day e-cigarette use with school-level prevalence of (i) e-cigarette use susceptibility (in never e-cigarette users; Supplementary Fig. 1A), (ii) cigarette smoking susceptibility (in never cigarette smokers; Supplementary Fig. 1B), (iii) ever cigarette smoking (Supplementary Fig. 1C), (iv) past 30-day cigarette smoking (Supplementary Fig. 1D), and (v) intention to quit cigarette smoking (in past 30-day cigarette smokers; Supplementary Fig. 1E) were all significant. The correlation with school-level prevalence of intention to quit cigarette smoking was relatively weak (r = 0.22).

Table 3 shows that the adjusted odds ratio (AOR) of e-cigarette use susceptibility in relation to high (vs low) school-level e-cigarette use was 1.40 (95% CI 1.05–1.87) in never e-cigarette users. The AORs of cigarette smoking susceptibility in relation to medium and high (vs low) school-level e-cigarette use were 1.24 (1.01–1.52) and 1.34 (1.02–1.75), respectively, in never cigarette smokers. In relation to per 1% increase in school-level e-cigarette use, the AORs of both susceptibility measures were significant.

**Table 3:** Association of school-level past 30-day e-cigarette use prevalence with student-level e-cigarette use and cigarette smoking susceptibility.

		School-level past 30-day e-cigarette use prevalence			
		Low^a^	Medium^a^	High^a^	Per 1% increase^b^
E-cigarette use susceptibility (in never e-cigarette users)	**CORs (95% CIs)**	1	1.20 (0.97–1.48)	1.54 (1.24–1.91)***	1.04 (1.02–1.07)***
	**AORs (95% CIs)** ^**c**^	1	1.15 (0.92–1.44)	1.40 (1.05–1.87)*	1.04 (1.00–1.07)*
Cigarette smoking susceptibility (in never cigarette smokers)	**CORs (95% CIs)**	1	1.39 (1.15–1.68)***	1.73 (1.43–2.11)***	1.06 (1.04–1.08)***
	**AORs (95% CIs)** ^**d**^	1	1.24 (1.01–1.52)*	1.34 (1.02–1.75)*	1.03 (1.00–1.07)*

^*^P < 0.05; **P < 0.01; ***P < 0.001.

CORs = crude odds ratios; AORs = adjusted odds ratios.

^a^Low: 0–1.99%; medium: 2.00–4.94%; high: 5.35–18.25%.

^b^Per 1% increase was per unit increase in school-level past 30-day e-cigarette use prevalence (continuous; range 0–18.25).

^c^With adjustment of age, sex, perceived family affluence, school-level past 30-day cigarette smoking prevalence (continuous), and cigarette smoking status.

^d^With adjustment of age, sex, perceived family affluence, school-level past 30-day cigarette smoking prevalence (continuous), and e-cigarette use status.

Table 4 shows that the AORs of ever cigarette smoking in relation to medium and high (vs low) school-level e-cigarette use were 1.83 (1.40–2.40) and 3.00 (2.28–3.93), respectively. The corresponding AORs of past 30-day cigarette smoking were 2.08 (1.48–2.94) and 3.55 (2.53–4.99). In relation to per 1% increase in school-level e-cigarette use, the AORs of ever and past 30-day cigarette smoking were both significant. However, in adjusted models, school-level e-cigarette use was not associated with intention to quit in past 30-day cigarette smokers.

**Table 4:** Association of school-level past 30-day e-cigarette use prevalence with student-level cigarette smoking and intention to quit.

		School-level past 30-day e-cigarette use prevalence			
		Low^a^	Medium^a^	High^a^	Per 1% increase^b^
Ever cigarette smoking	**CORs (95% CIs)**	1	2.72 (2.02–3.68)***	5.54 (4.10–7.49)***	1.17 (1.14–1.21)***
	**AORs (95% CIs)** ^**c**^	1	1.83 (1.40–2.40)***	3.00 (2.28–3.93)***	1.10 (1.07–1.14)***
Past 30-day cigarette smoking	**CORs (95% CIs)**	1	4.12 (2.90–5.86)***	10.84 (7.67–15.34)***	1.24 (1.20–1.29)***
	**AORs (95% CIs)** ^**c**^	1	2.08 (1.48–2.94)***	3.55 (2.53–4.99)***	1.11 (1.08–1.15)***
Intention to quit	**CORs (95% CIs)**	1	1.16 (0.67–1.98)	1.42 (0.85–2.39)	1.05 (1.00–1.09)*
(in past 30-day cigarette smokers)	**AORs (95% CIs)** ^**d**^	1	1.05 (0.60–1.82)	1.18 (0.65–2.13)	1.04 (0.99–1.10)

*P < 0.05; **P < 0.01; ***P < 0.001.

CORs = crude odds ratios; AORs = adjusted odds ratios.

^a^Low: 0–1.99%; medium: 2.00–4.94%; high: 5.35–18.25%.

^b^Per 1% increase was per unit increase in school-level past 30-day e-cigarette use prevalence (continuous; range 0–18.25).

^c^With adjustment of age, sex, per^c^eived family affluence, and e-cigarette use status.

^d^With adjustment of age, sex, perceived family affluence, school-level past 30-day cigarette smoking prevalence (continuous), and e-cigarette use status.

Table 5 shows that, in crude models, school-level e-cigarette use was generally associated with past 30-day use of all NCTPs. In adjusted model, however, all these associations became non-significant.

**Table 5:** Association of school-level past 30-day e-cigarette use prevalence with student-level past 30-day NCTP use^a^.

NCTPs		School-level past 30-day e-cigarette use prevalence			
		Low^b^	Medium^b^	High^b^	Per 1% increase^c^
Waterpipe	**CORs (95% CIs)**	1	2.07 (1.05–4.06)*	5.05 (2.75–9.28)***	1.25 (1.15–1.35)***
	**AORs (95% CIs)** ^**d**^	1	0.92 (0.44–1.94)	1.80 (0.86–3.76)	1.11 (0.99–1.24)
Chewing tobacco	**CORs (95% CIs)**	1	3.00 (0.71–12.71)	6.53 (1.70–25.02)**	1.31 (1.15–1.49)***
	**AORs (95% CIs)** ^**d**^	1	0.81 (0.22–3.04)	1.10 (0.29–4.11)	1.13 (0.96–1.33)
Cigar	**CORs (95% CIs)**	1	4.16 (1.44–12.03)**	7.59 (2.77–20.81)***	1.25 (1.10–1.43)**
	**AORs (95% CIs)** ^**d**^	1	1.20 (0.43–3.38)	1.52 (0.53–4.33)	0.96 (0.84–1.11)
Snus	**CORs (95% CIs)**	1	2.14 (0.91–5.02)	3.31 (1.52–7.19)**	1.21 (1.11–1.32)***
	**AORs (95% CIs)** ^**d**^	1	0.63 (0.23–1.72)	0.61 (0.22–1.74)	0.99 (0.85–1.15)
Smoking pipe	**CORs (95% CIs)**	1	1.79 (0.44–7.26)	4.19 (1.17–14.97)*	1.25 (1.06–1.46)**
	**AORs (95% CIs)** ^**d**^	1	0.56 (0.13–2.32)	0.83 (0.19–3.61)	1.03 (0.84–1.27)
Other tobacco products	**CORs (95% CIs)**	1	3.23 (1.14–9.15)*	3.68 (1.34–10.11)*	1.18 (1.03–1.35)*
	**AORs (95% CIs)** ^**d**^	1	1.33 (0.34–5.19)	0.95 (0.21–4.24)	0.95 (0.76–1.18)

*P < 0.05; **P < 0.01; ***P < 0.001.

CORs = crude odds ratios; AORs = adjusted odds ratios.

^a^NCTP = Non-cigarette tobacco product. The analysis was conducted in a subsample (10923 students; 30 schools) of the whole sample (40202 students; 92 schools). Only the subsample had data on NCTP use.

^b^Low: 0–2.00%; medium: 2.07–4.07%; high: 4.10–12.57%.

^c^Per 1% increase was per unit in^c^rease in school-level past 30-day e-cigarette use prevalence (continuous; range 0–12.57).

^d^With adjustment of age, sex, perceived family affluence, school-level past 30-day cigarette smoking prevalence (continuous), e-cigarette use status, and cigarette smoking status.

In general, school-level e-cigarette use was not associated with monthly, weekly, or daily drinking in adjusted models (Supplementary Table 1). An exception was a weak yet significant inverse association between school-level e-cigarette use (continuous) and monthly drinking. In the sensitivity analysis in which school-level past 30-day e-cigarette use (study factor in the main analysis) was replaced by school-level ever e-cigarette use, the results were similar with those of the main analysis (Supplementary Tables 2–4).

## Discussion

We found that, after covariate adjustment, school-level e-cigarette use (study factor) was strongly and linearly associated with e-cigarette use susceptibility in never e-cigarette users, and with cigarette smoking susceptibility in never cigarette smokers. Each 1% increase in school-level e-cigarette use was associated with 4% and 3% increase in the odds of being susceptible to use e-cigarettes and cigarettes, respectively. Reverse causality seemed unlikely because susceptibility to tobacco use, not being an easily observable phenotype, was unlikely to affect others’ behaviours.

After controlling for the same set of covariates, we found no positive association between the study factor and alcohol drinking (negative controls). Such findings do not support uncontrolled confounding effect on the association between school-level e-cigarette use and the two susceptibility measures, and thus lend credibility to causal interpretations. However, negative control analysis provides only suggestive evidence, and its value depends on the extent to which the two aforementioned selection criteria for negative controls are met^16^.

Qualitative data in US adolescents suggest that e-cigarette use in schools may be common because e-cigarette aerosol dissipates quickly and the device is easy to conceal^18^. There was even a US media report of adolescents using e-cigarettes in classrooms^19^. In a survey of US adult experienced e-cigarette users, almost two-thirds reported stealth vaping in places where e-cigarette use was prohibited^20^. Moreover, marketed as a product with many youth-appealing features^21^, e-cigarettes may be used by students to show off in front of schoolmates. The above all support that school-level e-cigarette use could have an impact on adolescents.

The observed associations of school-level e-cigarette use with e-cigarette use susceptibility were comparable with those of a US cross-sectional study^8^. In that study, medium and high (vs low) school-level e-cigarette use were, in never e-cigarette users, associated with the intention to use e-cigarettes soon (AORs 1.41 and 1.49) and if they were offered by friends (AORs 1.27 and 1.47). It is possible that school-level e-cigarette use influenced students’ normative beliefs and attitudes related to e-cigarettes, which in turn affected susceptibility to use e-cigarettes. Therefore, prevalent e-cigarette use in a school may lead to a snowball effect, in which an increasingly large number of students will become e-cigarette users. This is consistent with a US study showing that school-level clustering of e-cigarette use increased between 2011 and 2013^22^. E-cigarette use susceptibility was a surrogate of actual uptake behaviour. We collected past 30-day and ever e-cigarette use data but did not include them as outcomes, because they were the very variables that determined school-level e-cigarette use, so associations were predicted.

Our study is the first to show that school-level e-cigarette use was associated with cigarette smoking susceptibility in never cigarette smokers. This is consistent with the e-cigarette industry’s vision of using vaping to renormalise smoking^9^. It is possible that school environments with prevalent e-cigarette use normalised not only e-cigarette use but also “smoking-like” behaviours in general, and thus led students to be more susceptible to cigarette smoking. This effect, if confirmed, would represent a pathway by which e-cigarettes negatively affect population health.

We found that, after covariate adjustment, school-level e-cigarette use was not associated with intention to quit in past 30-day cigarette smokers. It is possible that school-level e-cigarette use encouraged only cigarette uptake but not cigarette smoking continuation. From another perspective, the absence of a positive association suggests that, although e-cigarettes were promoted as smoking cessation aids, their popularity in schools may not have increased quitting intentions. It is also possible that school-level e-cigarette use encouraged cigarette smoking continuation for some students and increased quitting intentions for others, and the overall effect could not be observed.

We found school-level e-cigarette use associated with ever and past 30-day cigarette smoking after covariate adjustment. These were in line with the association of school-level e-cigarette use with cigarette smoking susceptibility. It should be noted, however, that the AORs of ever and past 30-day cigarette smoking were controlled for fewer covariates than the AORs of cigarette smoking susceptibility. When the outcomes were cigarette smoking, we could not adjust for (or condition on a stratum of) cigarette smoking and school-level cigarette smoking prevalence. Ideally, these AORs should be estimated in longitudinal research in baseline never cigarette smokers, with adjustment of potential confounders.

We found that school-level e-cigarette use was not associated with past 30-day use of any of the NCTPs after covariate adjustment. This, in part, was inconsistent with individual-level longitudinal research which showed that e-cigarette use was associated with the uptake of waterpipe, cigars, and pipes^23^. One explanation is that our study had insufficient statistical power to detect associations when the outcomes were rare. Another explanation is that Hong Kong adolescents’ access to NCTPs was limited. It was also possible that school-level e-cigarette use only affected the use of combustible tobacco products, of which the look and use were similar with those of e-cigarettes.

Hong Kong is a setting where many tobacco control measures are already in place^24^. With low smoking prevalence and low exposure to the well-established risk factors (eg. tobacco advertising and unregulated cigarette packets)^12^, high school-level e-cigarette use may be associated with larger increases in tobacco use in Hong Kong students than in students in other settings where tobacco is more normalised.

Our study has several limitations. First, like all observational studies, confounding effect on the observed associations cannot be ruled out. Second, our study was weakened by the cross-sectional design. However, large samples are needed to ensure statistical power to study school-level influences, and large scale school-based follow-up surveys are costly and difficult. Third, our study was based on questionnaire survey data and may thus be affected by reporting bias. The measurement of our study factors (school-level e-cigarette use) may also involve error because they were based on aggregated student-level responses. Fourth, our survey had a low school-level response rate (36%). Nonetheless, the recruited and not recruited schools were similar in regard to districts, languages of instruction, sources of financial support, and single or mixed sex education (chi-square tests, ps > 0.05)^25^. Even if sample unrepresentativeness existed, it should not have affected the study of associations^26^.

Our study has several implications for research and practice. Our study is the first to bring attention to the potential effect of school-level e-cigarette use prevalence on cigarette smoking. Further studies of this effect should be conducted in different settings and using longitudinal designs. In addition, our findings are broadly in line with individual-level association of e-cigarette use with subsequent cigarette smoking^11^. Therefore, our findings add to the evidence that prevalent e-cigarette use in adolescents may increase cigarette smoking prevalence and support legislation to regulate e-cigarettes in adolescents. Lastly, although school teachers presumably disapprove student e-cigarette use, our findings highlight the importance of strictly banning e-cigarettes in schools. Given the well-established effect of school policies on student health behaviours^27^, such a ban may curb e-cigarette epidemics in adolescents, and may even help prevent potential reversal in the downward trend of cigarette smoking. School-level actions are particularly important in jurisdictions where government regulations are lagged behind.

To conclude, school-level e-cigarette use prevalence was associated with susceptibility to e-cigarette use and cigarette smoking, and ever and past 30-day cigarette smoking. The findings highlight the importance of strictly banning e-cigarettes in schools, and add to the evidence that prevalent e-cigarette use in adolescents may increase cigarette smoking prevalence.

## Supplementary information

Supplementary Info

## Data Availability

The datasets analysed during the current study are available from the corresponding author on reasonable request.
