# Supplementary material for: School-level electronic cigarette use prevalence and student-level tobacco use intention and behaviours
Source: Sci Rep. 2019 Feb 8;9:1690. doi: 10.1038/s41598-018-38266-z (PMC6368577; doi:10.1038/s41598-018-38266-z)
Supplement: Supplementary file 1 — Supplementary Info [file 41598_2018_38266_MOESM1_ESM.pdf]

# School-level electronic cigarette use prevalence and student-level tobacco use intention and behaviours

Jianjiu Chen[1], MPhil; Sai Yin Ho[1]*, PhD; Lok Tung Leung[1], MPhil; Man Ping Wang[2], PhD; Tai Hing Lam[1], MD

**Affiliations:** [1]School of Public Health, University of Hong Kong, Hong Kong Special Administrative Region, People's Republic of China; [2]School of Nursing, University of Hong Kong, Hong Kong Special Administrative Region, People's Republic of China

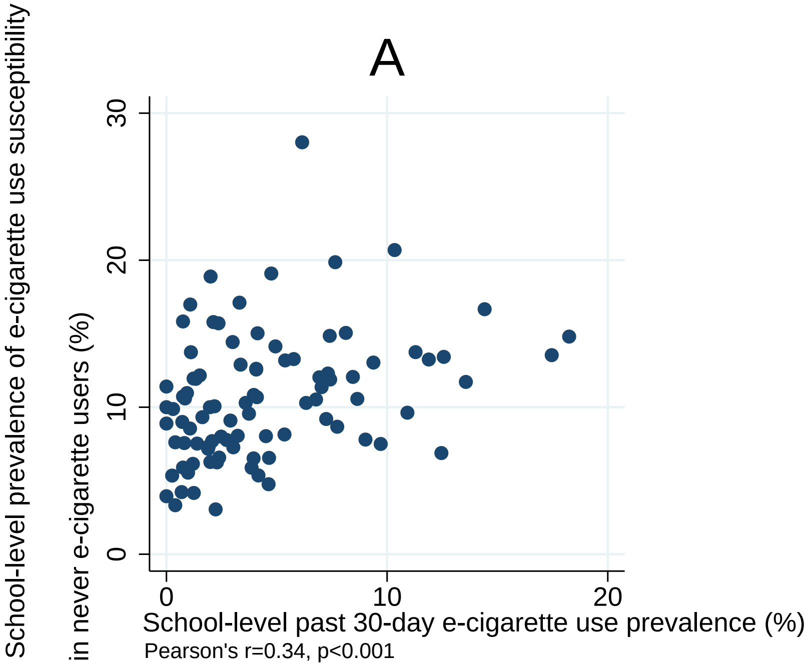

Pearson's r=0.34, p<0.001

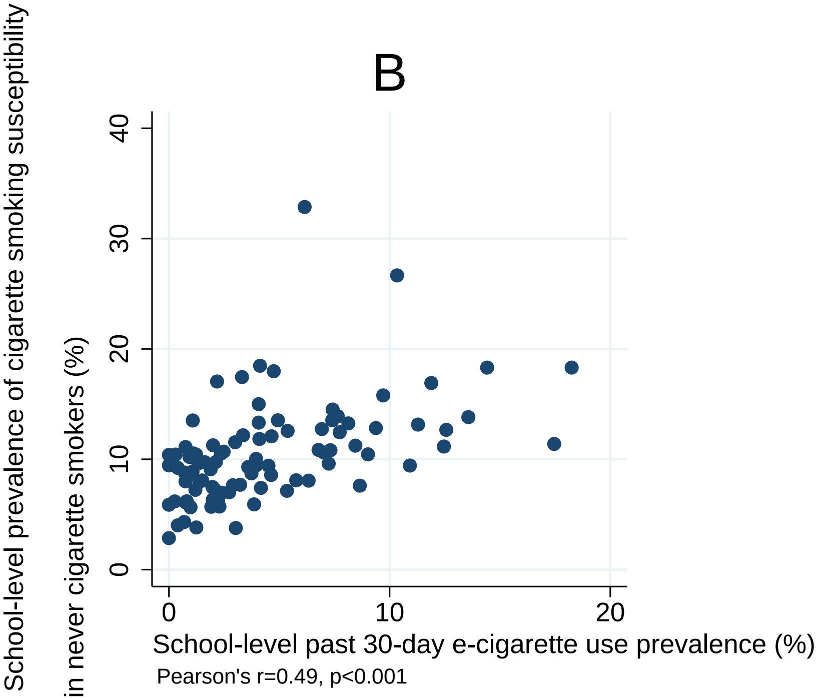
B
School-level prevalence of cigarette smoking susceptibility
in never cigarette smokers (%)
0
10
20
30
40
0
10
20
School-level past 30-day e-cigarette use prevalence (%)
Pearson's r=0.49, p<0.001

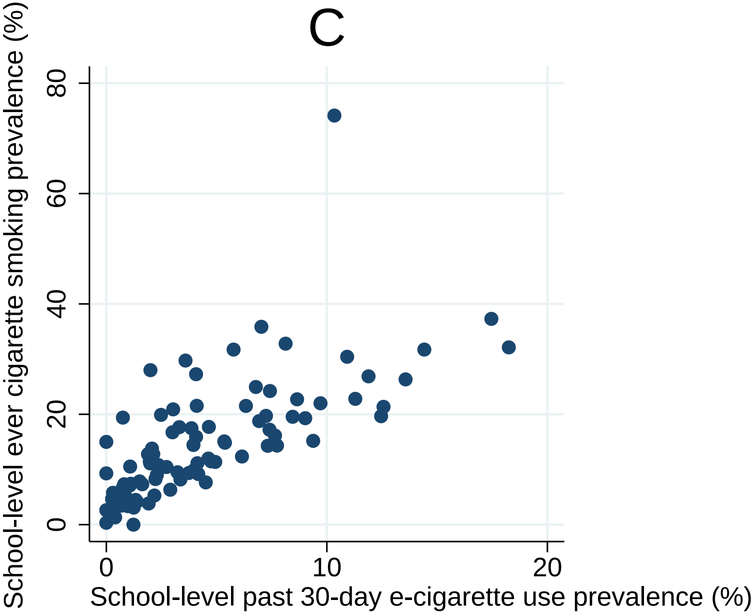


Pearson's r=0.70, p<0.001

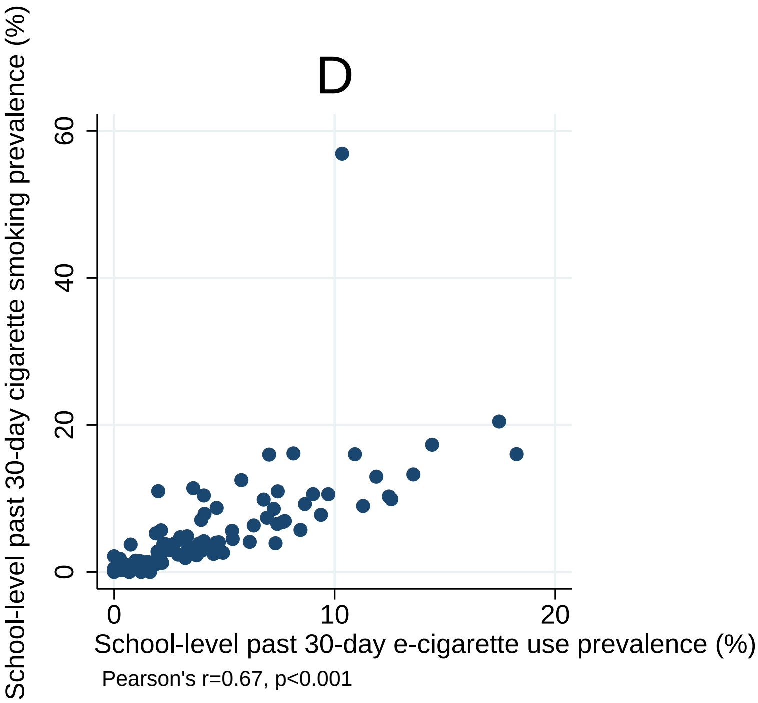
D
School-level past 30-day cigarette smoking prevalence (%)
0
20
40
60
0
10
20
School-level past 30-day e-cigarette use prevalence (%)
Pearson's r=0.67, p<0.001

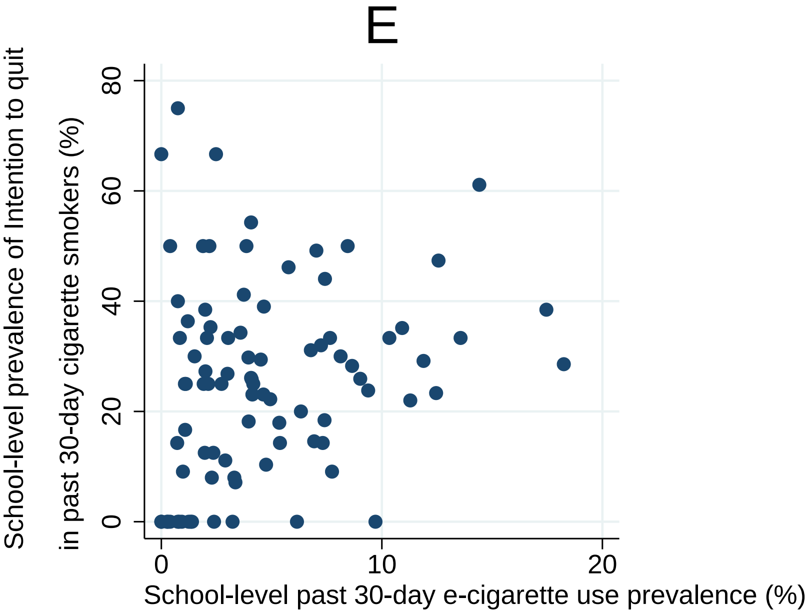


Pearson's r=0.22, p=0.04

**Supplementary Figure 1 (A-E). Correlation of school-level past 30-day e-cigarette use prevalence with school-level prevalence of other intention and behaviour indicators related to tobacco use**

**Supplementary Table 1. Association of school-level past 30-day e-cigarette use prevalence with student-level alcohol drinking**

| | | School-level past 30-day e-cigarette use prevalence | | | |
|---|---|---|---|---|---|
| | | **Low[a]** | **Medium[a]** | **High[a]** | **Per 1% increase[b]** |
| **Monthly drinking** | **CORs (95% CIs)** | 1 | 1.39 (1.20-1.61) *** | 1.66 (1.43-1.93) *** | 1.05 (1.04,1.07) *** |
| | **AORs (95% CIs)[c]** | 1 | 1.00 (0.88-1.15) | 0.88 (0.74-1.05) | 0.98 (0.96,1.00) * |
| **Weekly drinking** | **CORs (95% CIs)** | 1 | 1.95 (1.57-2.41) *** | 2.84 (2.29-3.51) *** | 1.11 (1.09,1.13) *** |
| | **AORs (95% CIs)[c]** | 1 | 1.15 (0.96-1.37) | 0.98 (0.79-1.22) | 1.00 (0.97,1.02) |
| **Daily drinking** | **CORs (95% CIs)** | 1 | 1.88 (1.22-2.91) ** | 3.62 (2.38-5.51) *** | 1.14 (1.09,1.18) *** |
| | **AORs (95% CIs)[c]** | 1 | 0.93 (0.61-1.40) | 0.86 (0.53-1.39) | 0.99 (0.94,1.04) |

*P < 0.05; **P < 0.01; ***P < 0.001.

CORs=crude odds ratios; AORs=adjusted odds ratios.

[a] Low: 0-1.99%; medium: 2.00-4.94%; high: 5.35-18.25%.

[b] Per 1% increase was per unit increase in school-level past 30-day e-cigarette use prevalence (continuous; range 0-18.25).

[c] With adjustment of age, sex, perceived family affluence, school-level past 30-day cigarette smoking prevalence (continuous), e-cigarette use status, and cigarette smoking status.

**Supplementary Table 2. Association of school-level ever e-cigarette use prevalence with student-level e-cigarette use and cigarette smoking susceptibility**

| | | School-level ever e-cigarette use prevalence | | | |
|---|---|---|---|---|---|
| | | **Low[a]** | **Medium[a]** | **High[a]** | **Per 1% increase[b]** |
| **E-cigarette use susceptibility** | **CORs (95% CIs)** | 1 | 1.31 (1.07-1.61) ** | 1.65 (1.34-2.04) *** | 1.03 (1.02-1.04) *** |
| **(in never e-cigarette users)** | **AORs (95% CIs)[c]** | 1 | 1.27 (1.02-1.58) * | 1.58 (1.20-2.08) ** | 1.05 (1.03-1.07) *** |
| **Cigarette smoking susceptibility** | **CORs (95% CIs)** | 1 | 1.42 (1.18-1.71) *** | 1.80 (1.49-2.18) *** | 1.04 (1.02-1.05) *** |
| **(in never cigarette smokers)** | **AORs (95% CIs)[d]** | 1 | 1.26 (1.02-1.54) * | 1.42 (1.09-1.85) * | 1.04 (1.02-1.06) *** |

*P < 0.05; **P < 0.01; ***P < 0.001.

CORs=crude odds ratios; AORs=adjusted odds ratios.

[a] Low: 0-4.88%; medium: 5.11-13.56%; high: 13.59-50.00%.

[b] Per 1% increase was per unit increase in school-level ever e-cigarette use prevalence (continuous; range 0-50.00).

[c] With adjustment of age, sex, perceived family affluence, school-level past 30-day cigarette smoking prevalence (continuous), and cigarette smoking status.

[d] With adjustment of age, sex, perceived family affluence, school-level past 30-day cigarette smoking prevalence (continuous), and e-cigarette use status.

**Supplementary Table 3. Association of school-level ever e-cigarette use prevalence with student-level cigarette smoking and intention to quit**

| | | School-level ever e-cigarette use prevalence | | | |
|---|---|---|---|---|---|
| | | **Low[a]** | **Medium[a]** | **High[a]** | **Per 1% increase[b]** |
| **Ever cigarette smoking** | **CORs (95% CIs)** | 1 | 3.11 (2.35-4.12) *** | 5.91 (4.46-7.83) *** | 1.10 (1.08-1.11) *** |
| | **AORs (95% CIs)[c]** | 1 | 2.01 (1.54-2.62) *** | 3.08 (2.36-4.03) *** | 1.06 (1.05-1.08) *** |
| **Past 30-day cigarette smoking** | **CORs (95% CIs)** | 1 | 4.52 (3.16-6.47) *** | 10.85 (7.59-15.50) *** | 1.12 (1.10-1.14) *** |
| | **AORs (95% CIs)[c]** | 1 | 2.19 (1.54-3.11) *** | 3.48 (2.44-4.95) *** | 1.06 (1.05-1.08) *** |
| **Intention to quit (in past 30-day** | **CORs (95% CIs)** | 1 | 1.50 (0.87-2.58) | 1.84 (1.09-3.13) * | 1.03 (1.01-1.05) ** |
| **cigarette smokers)** | **AORs (95% CIs)[d]** | 1 | 1.36 (0.78-2.38) | 1.60 (0.87-2.91) | 1.03 (0.99-1.07) |

*P < 0.05; **P < 0.01; ***P < 0.001.

CORs=crude odds ratios; AORs=adjusted odds ratios.

[a] Low: 0-4.88%; medium: 5.11-13.56%; high: 13.59-50.00%.

[b] Per 1% increase was per unit increase in school-level ever e-cigarette use prevalence (continuous; range 0-50.00).

[c] With adjustment of age, sex, perceived family affluence, and e-cigarette use status.

[d] With adjustment of age, sex, perceived family affluence, school-level past 30-day cigarette smoking prevalence (continuous), and e-cigarette use status.

**Supplementary Table 4. Association of school-level ever e-cigarette use prevalence with student-level past 30-day NCNE tobacco product use[a]**

| NCNE tobacco products (%)[b] | | School-level ever e-cigarette use prevalence | | | |
|---|---|---|---|---|---|
| | | Low[c] | Medium[c] | High[c] | Per 1% increase[d] |
| **Waterpipe** | **CORs (95% CIs)** | 1 | 4.11 (2.14-7.90) *** | 5.77 (3.00-11.12) *** | 1.14 (1.09-1.19) *** |
| | **AORs (95% CIs)[e]** | 1 | 1.84 (0.81-4.16) | 1.67 (0.63-4.43) | 1.05 (0.97-1.13) |
| **Chewing tobacco** | **CORs (95% CIs)** | 1 | 2.44 (0.65,9.15) | 9.16 (2.77-30.26) *** | 1.17 (1.06-1.28) ** |
| | **AORs (95% CIs)[e]** | 1 | 0.78 (0.19-3.24) | 1.83 (0.41,8.26) | 1.03 (0.92-1.16) |
| **Cigar** | **CORs (95% CIs)** | 1 | 3.20 (1.23,8.36) * | 7.24 (2.87-18.26) *** | 1.15 (1.06-1.24) *** |
| | **AORs (95% CIs)[e]** | 1 | 0.77 (0.26-2.28) | 1.06 (0.32-3.56) | 0.96 (0.88-1.06) |
| **Snus** | **CORs (95% CIs)** | 1 | 3.20 (1.33-7.73) ** | 5.38 (2.27-12.73) *** | 1.11 (1.05-1.17) *** |
| | **AORs (95% CIs)[e]** | 1 | 1.16 (0.40-3.40) | 1.15 (0.32-4.13) | 0.95 (0.86-1.05) |
| **Smoking pipe** | **CORs (95% CIs)** | 1 | 1.45 (0.40-5.24) | 6.09 (1.97-18.84) ** | 1.12 (1.02-1.24) * |
| | **AORs (95% CIs)[e]** | 1 | 0.43 (0.09-2.11) | 1.05 (0.19-5.63) | 0.95 (0.82-1.10) |
| **Other tobacco products** | **CORs (95% CIs)** | 1 | 4.21 (1.56-11.38) ** | 5.20 (1.90-14.26) ** | 1.11 (1.03-1.20) ** |
| | **AORs (95% CIs)[e]** | 1 | 2.22 (0.53,9.33) | 1.81 (0.31-10.44) | 1.02 (0.88-1.17) |

*P < 0.05; **P < 0.01; ***P < 0.001.

CORs=crude odds ratios; AORs=adjusted odds ratios.

[a] NCNE=Non-cigarette non-e-cigarette. The analysis was conducted in a subsample (10923 students; 30 schools) of the whole sample (40202 students; 92 schools). Only the subsample had data on NCNE tobacco product use.

[b] Proportion of product users in the subsample.

[c] Low: 0-5.12%; medium: 5.28-11.47%; high: 12.41-21.35%.

[d] Per 1% increase was per unit increase in school-level ever e-cigarette use prevalence (continuous; range 0-21.35).

[e] With adjustment of age, sex, perceived family affluence, school-level past 30-day cigarette smoking prevalence (continuous), e-cigarette use status, and cigarette smoking status.
